# Comprehensive Analysis of the *RAV* Gene Family and Its Expression Patterns Under PEG-Simulated Drought Stress in *Avena sativa*

**DOI:** 10.3390/biology15191749

**Published:** 2026-10-02

**Authors:** Shirui Xu, Mingchuan Ma, Zhang Liu, Lijun Zhang, Longlong Liu

**Affiliations:** 1 Center for Agricultural Genetic Resources Research, Shanxi Agricultural University, Taiyuan 030031, China; 2 Houji Laboratory in Shanxi Province, Center for Agricultural Genetic Resources Research, Shanxi Agricultural University, Taiyuan 030031, China; 3 Key Laboratory of Minor Crop Germplasm Innovation and Molecular Breeding (Co-Construction by Ministry and Province), Ministry of Agriculture and Rural Affairs, Taiyuan 030031, China

**Keywords:** RAV transcription factor, *Avena sativa*, genome-wide identification, drought stress, gene expression

## Abstract

The RAV transcription factors play crucial roles in plant growth, development, and stress responses. However, the *RAV* gene family has not been systematically characterized in oat. In this study, we performed a genome-wide identification and characterization of the *RAV* gene family in oat and investigated their expression patterns under PEG-simulated drought stress. A total of 12 *AsRAV* genes were identified in oat, which were distributed across six chromosomes. The *AsRAV* genes were classified into four groups, with members within the same group sharing similar conserved motifs. Promoter *cis*-element analysis identified various stress- and hormone-related elements. Synteny analyses suggested that both tandem and segmental duplication events played important roles in the expansion of the *AsRAV* gene family. Furthermore, the qRT-PCR analyses showed that nine *AsRAV* genes were significantly up-regulated under PEG-simulated drought stress, with *AsRAV1* showing the highest expression level. Subcellular localization analysis revealed that AsRAV1 is localized to both the nucleus and the cytoplasm. Collectively, our findings provide a comprehensive characterization of the *AsRAV* genes and lay a foundation for further functional studies on the drought stress response in oat.

## Simple Summary

The RAV (related to ABI3/VP1) transcription factors regulate physiological and developmental processes in various plant species. However, little is known about the *RAV* gene family in oat (*Avena sativa* L.). In this study, we identified 12 *RAV* genes in oat and analyzed their genomic and evolutionary features. Expression pattern analysis revealed that nine *AsRAV* genes were significantly up-regulated under PEG-simulated drought stress, with *AsRAV1* showing the highest induction level. These findings provide basic information for further investigation of *AsRAV* genes under drought stress.

## 1. Introduction

As sessile organisms, plants are constantly exposed to a variety of environmental stresses during their life cycle. To cope with these challenges, they have evolved sophisticated transcriptional regulatory networks, in which the AP2/ERF and B3 transcription factors are recognized as crucial players in stress responses [1,2]. The RAV (related to ABI3 and VP1) transcription factors, which contain an N-terminal AP2 DNA-binding domain and a C-terminal B3 DNA-binding domain, represent a unique subfamily that is classified into both the AP2/ERF and B3 families [3]. Accordingly, RAV proteins can recognize the CACCTG motif via the B3 domain and the CAACA motif via the AP2 domain [4,5]. MeRAV proteins exhibit transcriptional activation activity by specifically binding to both the CAACA and CACCTG motifs [6]. FaRAV1 positively regulates anthocyanin accumulation in strawberry by directly binding to a CAACA motif in the *FaMYB10* promoter [7].

The *RAV* gene family plays important roles in plant physiological and developmental processes [8]. For instance, *GmRAV* represses the transcription of *GmGA3ox*, thereby reducing soybean plant height [9]. *CmTEM1*, a chrysanthemum homolog of the *Arabidopsis* flowering repressor *AtTEM1*, is involved in ethylene-regulated flowering and delays floral transition in chrysanthemum [4,10]. In addition, *RAVs* are involved in plant responses to environmental stresses including biotic and abiotic stresses. Overexpression of soybean *GmRAV-03* in *Arabidopsis* enhances salt and drought tolerance and confers insensitivity to exogenous ABA [11]. *CsRAV1* enhances NaCl and ABA tolerance in cucumber [12]. Overexpression of *GeRAV1* from *Gelsemium elegans* enhances the tolerance of *Escherichia coli* Rosetta cells to cold and NaCl [13]. *StHRAV1* has been identified as a negative regulator of thermotolerance in potato [14].

Oat (*Avena sativa* L.) is an allohexaploid (2*n* = 6*x* = 42, AACCDD) belonging to the family Poaceae. It is widely recognized for its rich content of bioactive compounds, including β-glucan and avenanthramides, which exhibit cholesterol-lowering and antioxidant activities [15,16]. Drought stress severely impairs the growth and development of oat, leading to significant reductions in biomass and grain yield [17,18]. Although RAV transcription factors have been reported to function in stress responses in various plant species, their involvement in drought stress tolerance in oat remains largely unexplored. In this study, we identified 12 *AsRAV* genes in oat and systematically analyzed their physicochemical properties, phylogenetic relationships, chromosomal locations, gene structures, conserved domains, *cis*-acting elements, synteny and selection pressure. Additionally, the expression patterns of *AsRAVs* in response to PEG-simulated drought stress were examined. These findings lay the foundation for classification and drought-responsive characterization of the *RAV* gene family in oat.

## 2. Materials and Methods

### 2.1. Identification and Characterization of RAV Genes in Oat

To identify RAV transcription factors in oat, the genome assembly and annotation of *Avena sativa* cv. Sang v1.1 were downloaded from the GrainGenes database (https://wheat.pw.usda.gov/GG3/content/avena-sang-download) (accessed on 22 October 2024) [19]. Putative *RAV* genes were identified through a combined BLASTP and Hidden Markov Model (HMM) search. For BLASTP, six *Arabidopsis thaliana* and four *Oryza sativa* RAV protein sequences were used as queries against the oat genome (Table S1) [3,20]. Concurrently, HMM profiles of the B3 (PF02362) and AP2 (PF00847) domains were utilized to screen for *RAV* candidates in oat [21]. All predicted genes were further validated for the presence of both B3 and AP2 domains using the Pfam (http://pfam.xfam.org/) (accessed on 9 July 2026) [22], NCBI-CDD (https://www.ncbi.nlm.nih.gov/Structure/cdd/wrpsb.cgi) (accessed on 9 July 2026) [23], and SMART databases (https://smart.embl.de/) (accessed on 9 July 2026) [24]. In addition, *RAV* gene family members in *Hordeum vulgare*, *Setaria italica*, and *Zea mays* were identified using the same method as described above for subsequent analyses. The amino acid length, molecular weight (MW), theoretical isoelectric point (pI), instability index, and grand average of hydropathy (GRAVY) of the AsRAV proteins were calculated using TBtools v2.423 [25].

### 2.2. Conserved Motifs, Gene Structures, and Cis-Acting Elements Analysis

Conserved motifs of AsRAV proteins were identified using MEME online tool (https://meme-suite.org/meme/tools/meme) (accessed on 10 July 2026) [26], with the maximum number of motifs set to 10, motif width limits of 6–50 amino acids, and zero or one occurrence per sequence (zoops mode). Gene structure analysis was performed using the GFF3 annotation of *A. sativa*. The 2000 bp sequences upstream of the translation start codon (ATG) of each *AsRAV* gene were extracted for *cis*-acting element prediction using the PlantCARE database (https://bioinformatics.psb.ugent.be/webtools/plantcare/html/) (accessed on 11 July 2026) [27]. Conserved motifs, gene structures, and *cis*-acting elements were visualized using TBtools.

### 2.3. Phylogenetic Tree

Phylogenetic analysis was performed using RAV protein sequences from *A. sativa*, *A. thaliana* (6) (Table S1) [3], *O. sativa* (4) (Table S1) [20], and *Triticum aestivum* (26) (Table S1) [21], *H. vulgare* (9) (Table S1), *S. italica* (4) (Table S1), and *Z. mays* (5) (Table S1), all of which contained both AP2 and B3 domains. A phylogenetic tree was constructed using MEGA 7.0 with the Neighbor-Joining method and 1000 bootstrap replicates. The tree was visualized and annotated using the Interaction Tree of Life (iTOL) (https://itol.embl.de/) (accessed on 16 July 2026) [28].

### 2.4. Chromosome Location, Gene Duplication Events, and Selection Pressure Analysis

All *AsRAV* genes were mapped to oat chromosomes based on the GFF3 annotation. Gene duplication and collinearity analyses of *AsRAV* genes were performed using TBtools. Genome sequences and annotation files of *T. aestivum* (IWGSC RefSeq v2.1), *O. sativa* (IRGSP-1.0), *H. vulgare* (MorexV3), *S. italica* (v2.0), *Z. mays* (B73 RefGen v5), and *A. thaliana* (TAIR10) were downloaded from the Ensembl Plants database (https://plants.ensembl.org/) (accessed on 21 September 2026). Syntenic analysis between oat and each of the five species was performed using the One Step MCScanX module in TBtools with an E-value threshold of 1 × 10^−10^ and a minimum of 5 BLAST hits (Num of blasthits = 5). For each *AsRAV* gene, the syntenic block with the smallest e-value was selected independently in each species. Up to five flanking genes on each side of the *AsRAV* gene were extracted from the selected block as co-separated genes. If fewer genes were available in a given block, all available genes were included. If no syntenic block was detected for a given *AsRAV* gene in a species, the corresponding entry was marked as absent. Non-synonymous (*Ka*) and synonymous (*Ks*) ratios for each duplicated gene pair were calculated using TBtools.

### 2.5. Plant Materials and Drought Stress Treatment

Hexaploid oat cultivar ‘Pinyan No. 8’, which showed good drought tolerance and agronomic performance in the field, was used in this study [29]. Seeds were surface-sterilized with 1.0% sodium hypochlorite for 20 min and then rinsed thoroughly with sterile distilled water. The sterilized seeds were germinated in Petri dishes, with 100 seeds per dish. After germination, seedlings were transferred to hydroponic boxes and cultivated in modified Hoagland nutrient solution in a greenhouse under a 16 h light/8 h dark photoperiod at 24 °C (day)/18 °C (night), 60% relative humidity, and a light intensity of 250 μmol m^−2^ s^−1^. Each treatment was performed with three biological replicates. At the one-leaf stage, seedlings were subjected to PEG-simulated drought stress by adding polyethylene glycol 6000 (PEG 6000) to the modified Hoagland nutrient solution to a final concentration of 15% (*w*/*v*), which generated an osmotic potential ranging from −0.30 to −0.33 MPa under the experimental temperature conditions [30,31]. Root samples were collected at 0, 6, 12, 24, 48, and 72 h after stress treatment, with three biological replicates.

### 2.6. Transcriptome Analysis of AsRAV Genes

Transcriptome data of oat under drought stress were retrieved from the NCBI Sequence Read Archive (accession no. SRP237902), which contains three biological replicates per time point. Differential expression was assessed with DESeq2 (v1.52.0) using raw read counts, and *p* values were adjusted by the Benjamini–Hochberg method. Log2 (fold changes) (log2 FC) were calculated at each time point (6, 12, 24, 48, and 72 h) relative to 0 h. Genes with padj < 0.05 and |log2 FC| ≥ 1 were considered differentially expressed.

### 2.7. qRT–PCR Analysis

Total RNA was extracted from root samples using the DP432 Kit (Tiangen, Beijing, China). First-strand cDNA was synthesized with the HiScript IV All-in-One Ultra RT SuperMix (Vazyme, Nanjing, China). qRT-PCR was performed using the FastReal qPCR PreMix (Tiangen, China) with AsActin as the reference control. Relative gene expression was calculated using the 2^−ΔΔCt^ method [32]. All reactions were performed in three biological replicates. The primer sequences are listed in Table S2.

### 2.8. Subcellular Localization

To determine the subcellular localization of AsRAV1, the full-length cDNA of *AsRAV1* was cloned into the *pCAMBIA1302-GFP* vector to generate the *AsRAV1-GFP* fusion construct. The recombinant plasmid was co-infiltrated with a nuclear marker (*NLS-mCherry*) into *Nicotiana benthamiana* leaves at a 1:1 ratio [33]. A free-GFP construct was used as a control. GFP and mCherry fluorescence were examined using confocal laser scanning microscopy (LSM800, Carl Zeiss, Oberkochen, Germany), and each experiment was performed three times.

### 2.9. Statistical Analysis

Statistical analyses were performed using GraphPad Prism 8.0. Significant differences (*p* < 0.05) were determined by one-way ANOVA followed by Tukey’s multiple comparison test. Data are presented as mean ± standard error of the mean (SEM). All graphs were generated using GraphPad Prism 8.0.

## 3. Results

### 3.1. Identification and Physicochemical Properties of RAV Genes in Oat

A total of 12 *AsRAV* genes were identified in the oat genome, all of which encode proteins containing conserved AP2 and B3 domains and were designated *AsRAV1* to *AsRAV12* (Table 1 and Table S3, Figure S1). The protein lengths of AsRAVs ranged from 321 to 477 aa, and the molecular weights ranged from 34.88 to 48.03 kDa. Among them, AsRAV7 had the shortest length (321 aa) and the lowest molecular weight (34.88 kDa), whereas AsRAV12 exhibited the longest length (477 aa) and the highest molecular weight (48.03 kDa). The pI of AsRAV3, AsRAV7, and AsRAV10 were below 7.00, and their instability indices were below 40, indicating that these three proteins are acidic and likely stable. In contrast, the remaining AsRAV proteins are basic and likely unstable. All AsRAV proteins had GRAVY below 0, indicating that these proteins are hydrophilic.

### 3.2. Phylogenetic Analysis of RAV Proteins from A. sativa, A. thaliana, O. sativa, T. aestivum, H. vulgare, S. italica, and Z. mays

**Figure 1:**
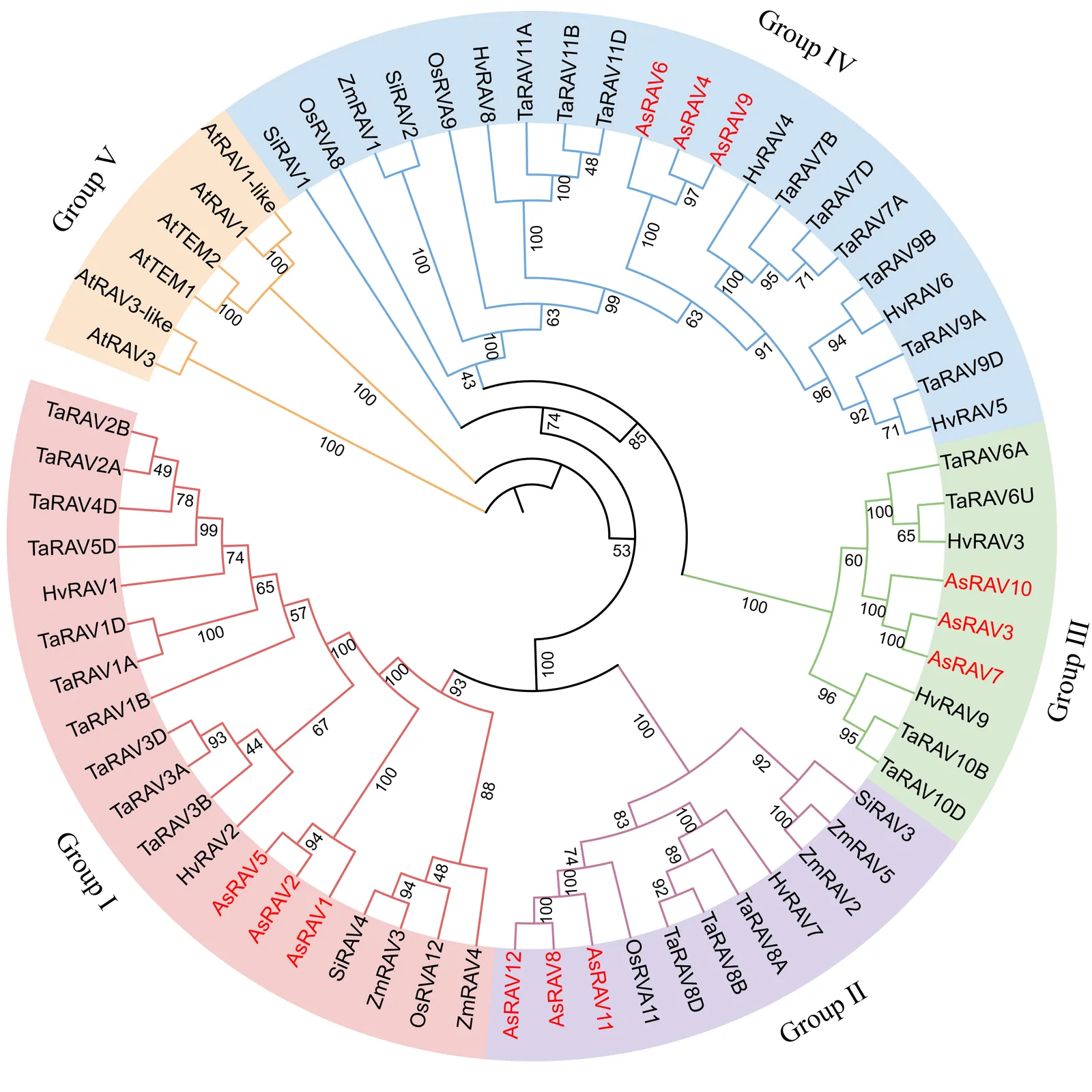
Phylogenetic relationships of RAV proteins in *A. sativa* (As), *A. thaliana* (At), *O. sativa* (Os), *T. aestivum* (Ta), *H. vulgare* (Hv), *S. italica* (Si), and *Z. mays* (Zm). The phylogenetic tree was constructed using the Neighbor-Joining method with 1000 bootstrap replicates. The five groups are distinguished by different colors.

To explore the evolutionary relationships of *RAV* genes among *A. sativa* (12), *A. thaliana* (6), *O. sativa* (4), *T. aestivum* (26), *H. vulgare* (9), *S. italica* (4), and *Z. mays* (5), the 66 RAV protein sequences were used to construct a phylogenetic tree (Figure 1). Phylogenetic analysis showed that RAV proteins were divided into five groups (Groups I–V). Group IV contained the most members (21), followed by Group I (19), Group II (11), Group III (9), and Group V (6). Notably, Groups I–IV each comprised three AsRAV proteins, along with variable numbers of RAV proteins from other species. In contrast, Group V contained only *A. thaliana* RAV proteins, with no AsRAV members identified.

### 3.3. Conserved Motif and Gene Structure Analysis of AsRAV Genes

**Figure 2:**
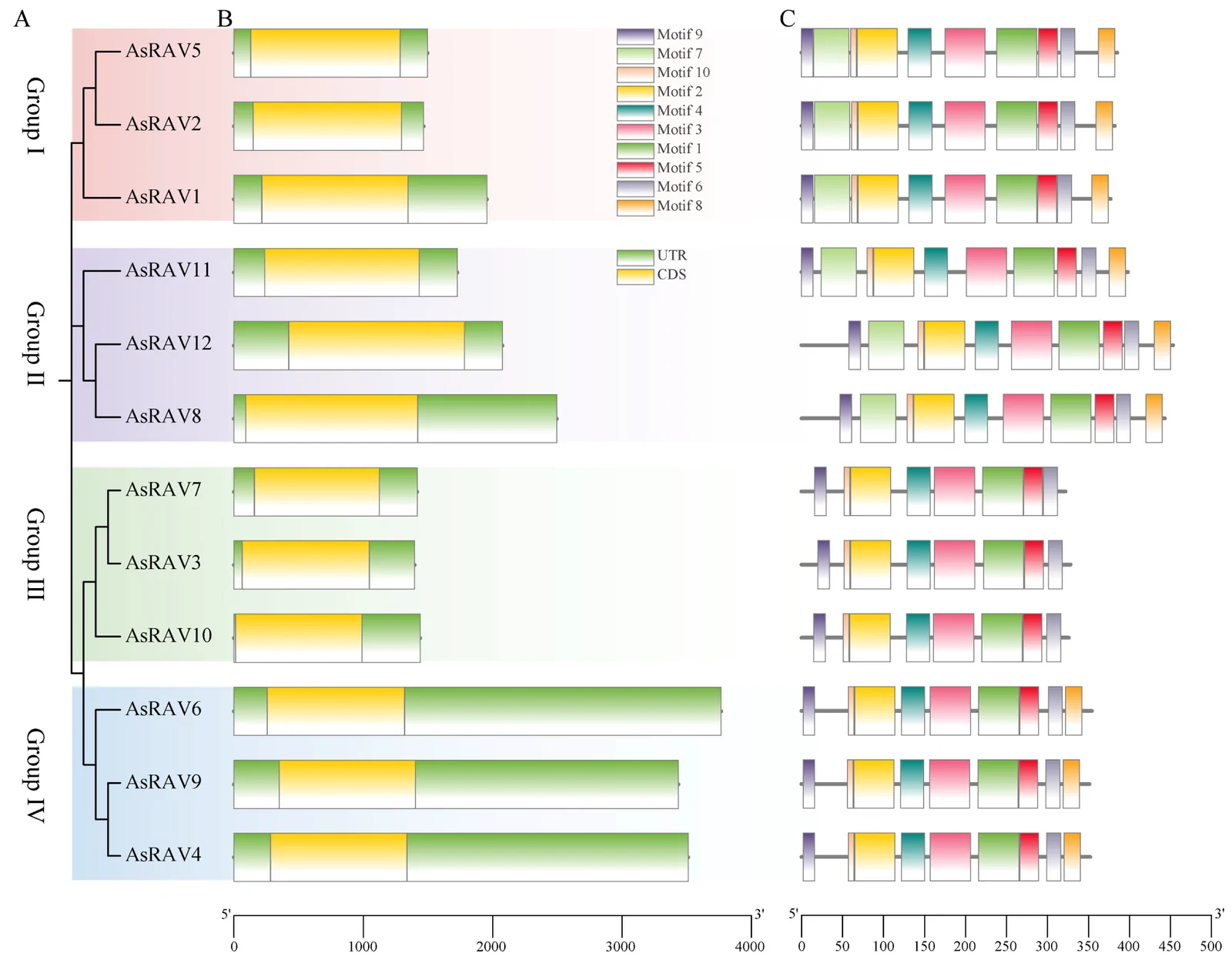
Phylogenetic relationships, gene structures, and conserved motifs of *AsRAVs*. (**A**) Phylogenetic tree of AsRAV proteins. Different groups are indicated by different colors. (**B**) Gene structure of *AsRAVs*. (**C**) Conserved motifs of AsRAV proteins. Different motifs are represented by different colored boxes.

Based on the phylogenetic tree, the 12 AsRAV proteins were classified into four groups (Groups I–IV) (Figure 2A). Gene structure analysis revealed that all *AsRAV* genes contain a single exon and lack introns (Figure 2B). Conserved motif analysis revealed that Group I and II proteins contained all ten motifs; Group III proteins lacked motifs 7 and 8; and Group IV proteins lacked only motif 7. All AsRAV proteins shared motifs 1, 2, 3, 4, 5, 6, 9, and 10, suggesting these motifs are highly conserved in AsRAV (Figure 2C and Figure S2). These group-specific motif variations imply potential functional divergence during evolution.

### 3.4. Chromosomal Distribution and Intraspecific Synteny Analysis of AsRAV Genes

**Figure 3:**
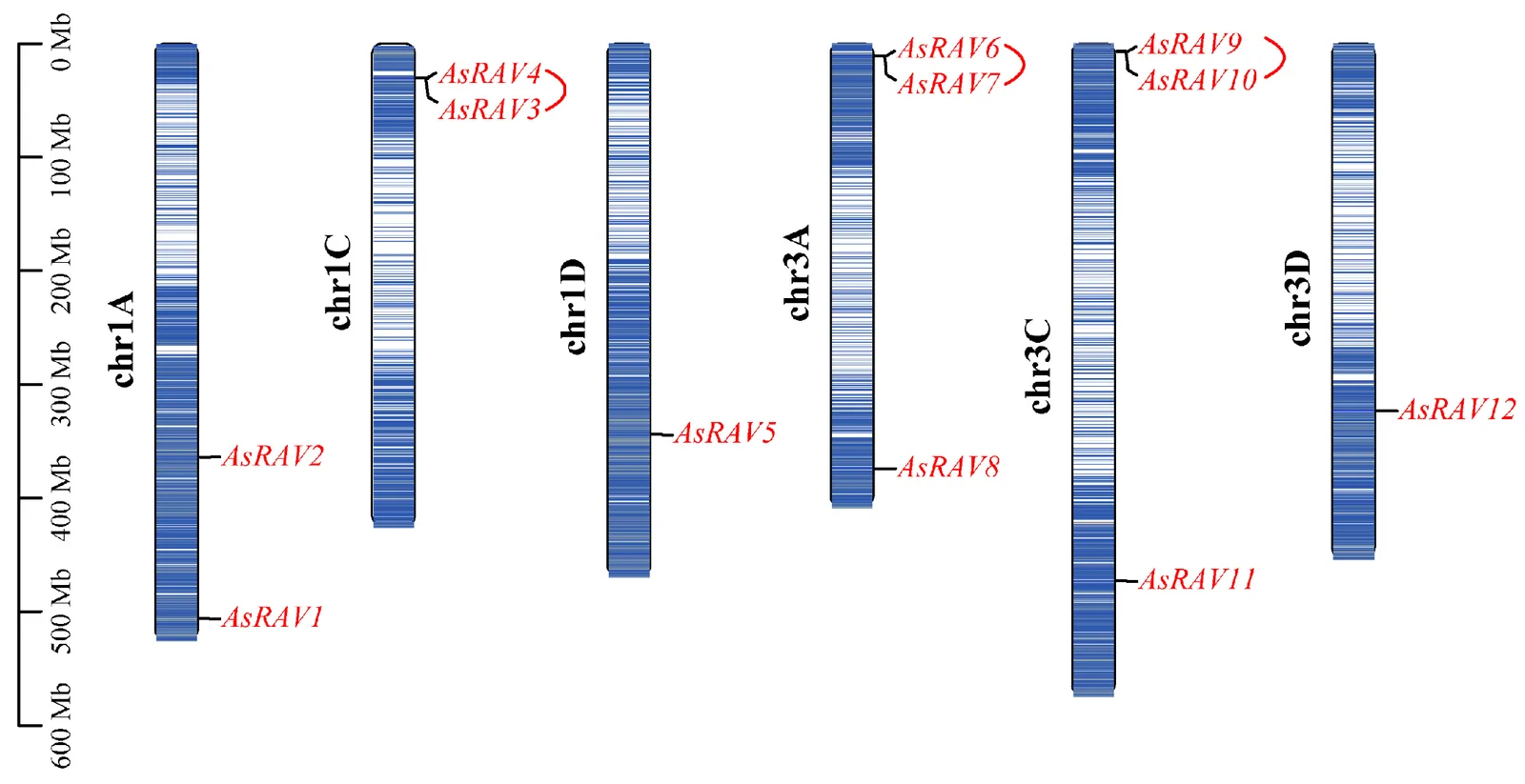
Chromosomal locations of *AsRAV* genes on six oat chromosomes. Tandem duplicated gene pairs are connected by curved red lines.

The 12 *AsRAV* genes were distributed on chromosomes 1A, 1C, 1D, 3A, 3C, and 3D of oat (Figure 3). Chromosomes 1D and 3D each contained one *AsRAV* gene; chromosomes 1A and 1C each contained two; and chromosomes 3A and 3C each contained three. There was no positive correlation between chromosome lengths and the number of *AsRAV* genes. In addition, the A subgenome contained *AsRAV1*, *AsRAV2*, *AsRAV6*, *AsRAV7*, and *AsRAV8*; the C subgenome contained *AsRAV3*, *AsRAV4*, *AsRAV9*, *AsRAV10*, and *AsRAV11*; and the D subgenome contained *AsRAV5* and *AsRAV12*, indicating an uneven distribution among the three subgenomes.

**Figure 4:**
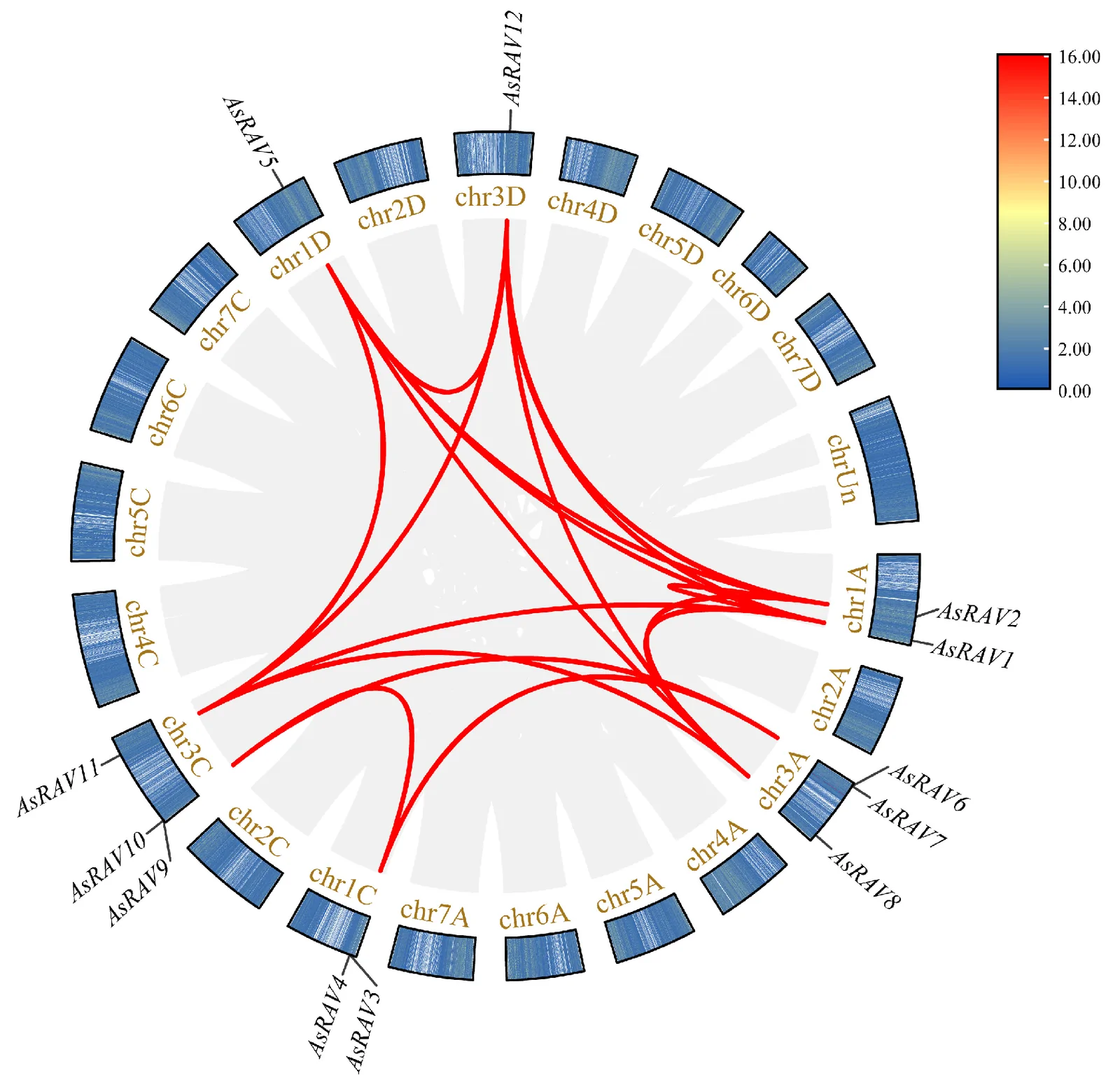
Intraspecific collinearity analysis of the *AsRAV* gene family. Gray lines represent syntenic regions within the oat genome. The red lines indicate a segmental duplication gene pair.

To understand the evolutionary expansion of the *AsRAV* gene family in oat, we performed an intraspecific synteny analysis. The collinearity analysis identified 21 duplication events (Figure 4, Table S4). Six *AsRAV* genes (*AsRAV3/4*, *AsRAV6/7*, and *AsRAV9/10*) were clustered into three tandem duplication event regions on chromosomes 1C, 3A, and 3C (Figure 3, Table S4). Additionally, 18 segmental duplication events with 11 *AsRAV* genes were identified. These results suggest that tandem and segmental duplications are involved in the expansion of the *AsRAV* gene family, with segmental duplication serving as the major driving force. Furthermore, the *Ka/Ks* ratios of all duplicated gene pairs were less than 1, ranging from 0.15 to 0.46 (Table S5), implying that the *AsRAV* genes have undergone purifying selection during evolution.

### 3.5. Interspecific Synteny Analysis of AsRAV Genes

**Figure 5:**
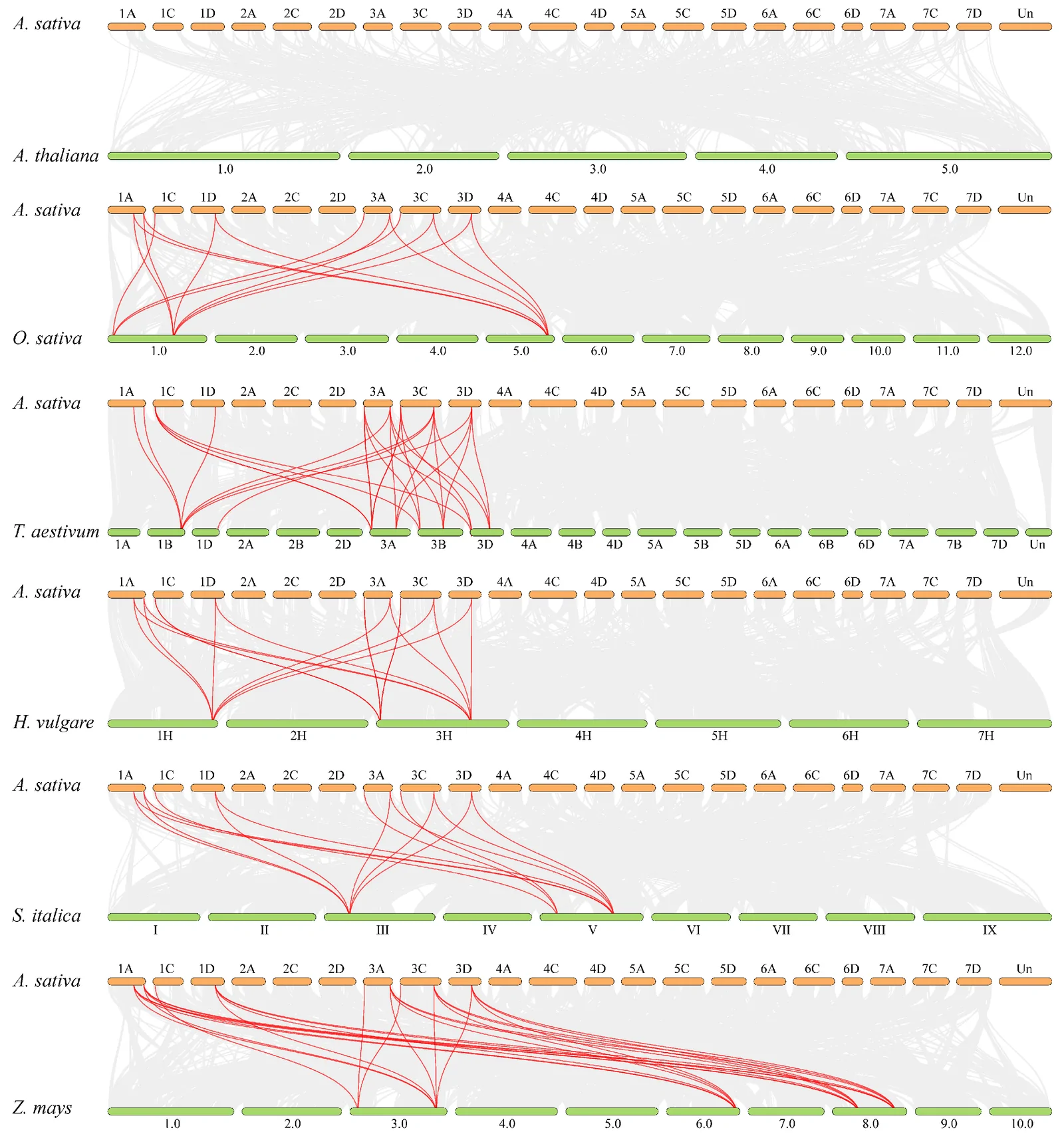
Syntenic relationships of *RAV* genes among *A. sativa*, *A. thaliana*, *O. sativa*, *T. aestivum*, *H. vulgare*, *S. italica*, and *Z. mays*. The gray lines indicate syntenic blocks within the genomes, and the red lines represent syntenic *RAV* gene pairs.

To further investigate the phylogenetic mechanisms of the *AsRAV* gene family, we constructed comparative syntenic maps between oat and six other species (*O. sativa*, *T. aestivum*, *H. vulgare*, *S. italica*, *Z. mays*, and *A. thaliana*) (Figure 5). Six *AsRAV* genes (*AsRAV1*, *AsRAV2*, *AsRAV5*, *AsRAV8*, *AsRAV11*, and *AsRAV12*) formed 12 orthologous gene pairs with two *OsRAV* genes (*OsRAV11* and *OsRAV12*), with each *AsRAV* gene being paired with two *OsRAV* genes (Table S6). Twelve *AsRAV* genes formed 28 orthologous gene pairs with 11 *TaRAV* genes (*TaRAV1B*, *TaRAV3B*, *TaRAV5D*, *TaRAV6A*, *TaRAV7A*, *TaRAV7B*, *TaRAV7D*, *TaRAV8A*, *TaRAV8B*, *TaRAV8D*, and *TaRAV9D*), with each *AsRAV* gene being paired with one to five *TaRAV* genes (Table S7). In addition, 12 *AsRAV* genes formed 18 orthologous gene pairs with four *HvRAV* genes (*HvRAV1*, *HvRAV3*, *HvRAV4*, and *HvRAV7*) (Table S8), 15 orthologous gene pairs with three *SiRAV* genes (*SiRAV2*, *SiRAV3*, and *SiRAV4*) (Table S9), and 27 orthologous gene pairs with five *ZmRAV* genes (*ZmRAV1*, *ZmRAV2*, *ZmRAV3*, *ZmRAV4*, and *ZmRAV5*) (Table S10). The *Ka/Ks* ratios of all orthologous pairs between oat and these species were less than 1 (Tables S6–S10), indicating that these *RAV* genes have undergone strong purifying selection during evolution. In contrast, no syntenic gene pairs were detected between *AsRAV* genes and *A. thaliana RAV* genes under the alignment criteria used in this study, suggesting a more distant evolutionary relationship between these genes.

To further examine whether the *AsRAV* genes are located in conserved genomic regions, we analyzed the co-separated genes within the syntenic blocks containing *AsRAV* genes across five grass species (Table S11). Among the 12 *AsRAV* genes examined, six (*AsRAV1*, *AsRAV2*, *AsRAV5*, *AsRAV8*, *AsRAV11*, and *AsRAV12*) were found within syntenic blocks in all five grass species, suggesting that these genes and their flanking regions may be relatively conserved. Three genes (*AsRAV4*, *AsRAV6*, and *AsRAV9*) showed synteny in four of the five species but not in rice, while three genes (*AsRAV3*, *AsRAV7*, and *AsRAV10*) were only detected in wheat and barley. These differences may reflect gene loss, genomic rearrangement, or other evolutionary events that occurred after species divergence.

### 3.6. Promoter Cis-Element Analysis of AsRAV Genes

**Figure 6:**
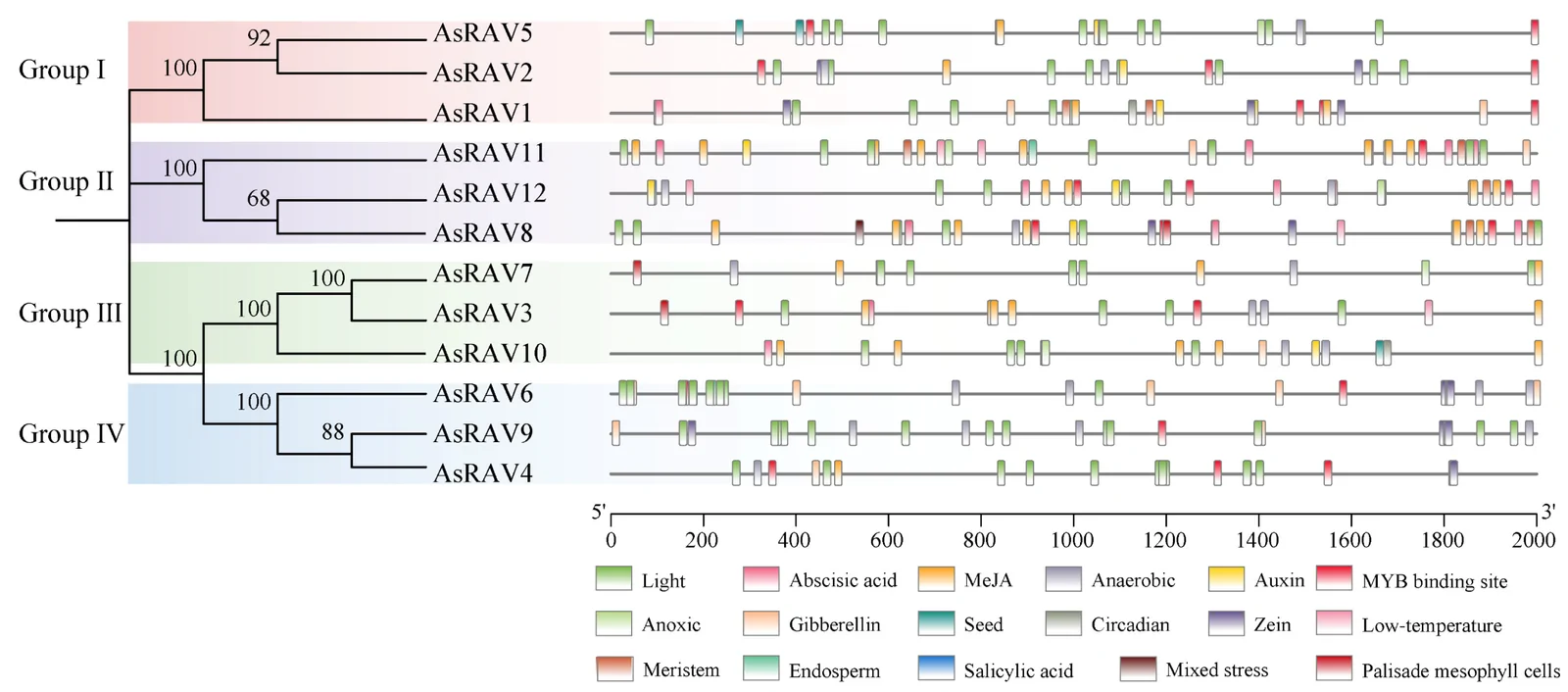
Promoter analysis of the *AsRAV* genes in oat. Boxes in different colors represent different *cis*-elements.

To explore the potential regulatory characteristics of *AsRAV* genes, we analyzed the *cis*-acting elements in their promoter regions. A total of 17 *cis*-elements were identified (Figure 6 and Figure S3). Notably, all *AsRAV* promoters contained light-responsive elements. ABA-responsive elements, anaerobic-responsive elements, MeJA-responsive elements, and MYB-binding sites were also widely distributed, occurring in 91.67% (11/12), 83.33% (10/12), 83.33% (10/12), and 83.33% (10/12) of *AsRAV* promoters, respectively. In addition, auxin-responsive elements were identified in 58.33% (7/12) of the promoters, gibberellin- and zein metabolism-responsive elements each in 50.00% (6/12), and anoxic-responsive elements in 41.67% (5/12). Low-temperature- and meristem-specific elements were detected in 33.33% (4/12) of the promoters, while the remaining elements were present in three or fewer promoters. Collectively, these predicted *cis*-elements represent putative regulatory features and suggest that *AsRAV* genes may be subject to regulation by multiple hormones and environmental factors.

### 3.7. Expression Pattern of AsRAV Genes Under PEG-Simulated Drought Stress

**Figure 7:**
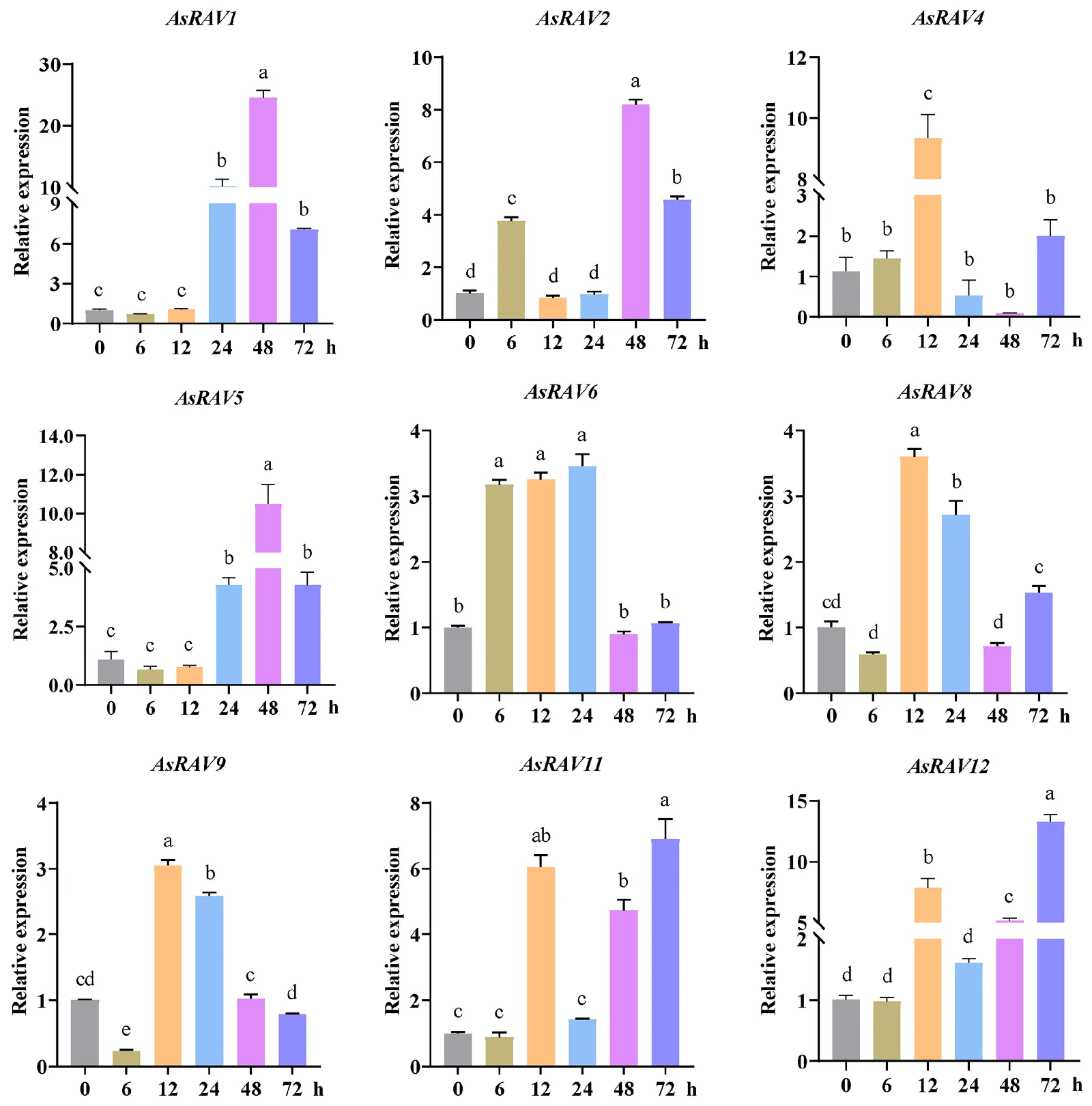
Relative expression of *AsRAV* genes in response to 15% PEG 6000 treatment. Error bars indicate standard error of the mean (SEM) of three independent experiments (*n* = 3 biological replicates). Significant differences (*p* < 0.05) were determined by one-way ANOVA with Tukey’s test and are indicated by different lowercase letters. Letters were calculated independently for each gene across the six time points.

To investigate the expression patterns of the 12 *AsRAV* genes under drought stress, transcriptomic analysis was performed using publicly available RNA-seq data across six time points (0, 6, 12, 24, 48, and 72 h) (Figure S4, Table S12). Nine *AsRAV* genes (*AsRAV1*, *AsRAV2*, *AsRAV4*, *AsRAV5*, *AsRAV6*, *AsRAV8*, *AsRAV9*, *AsRAV11*, and *AsRAV12*) were upregulated at specific time points (padj < 0.05, |log2FC| ≥ 1), while *AsRAV3* was downregulated at 48 h, and *AsRAV7* and *AsRAV10* showed no significant changes. Subsequently, the nine upregulated genes were selected for further validation by qRT-PCR under PEG-simulated drought treatment (Figure 7). The qRT-PCR results confirmed their drought-inducible expression, with distinct temporal patterns: *AsRAV1* and *AsRAV5* were upregulated at 24, 48, and 72 h; *AsRAV2* at 6, 48, and 72 h; *AsRAV4* at 12 h; *AsRAV6* at 6, 12, and 24 h; *AsRAV8* and *AsRAV9* at 12 and 24 h; and *AsRAV11* and *AsRAV12* at 12, 48, and 72 h. Collectively, these results suggest that the most *AsRAV* genes may be involved in the drought stress response in oat.

### 3.8. Subcellular Localization of AsRAV1 Protein

**Figure 8:**
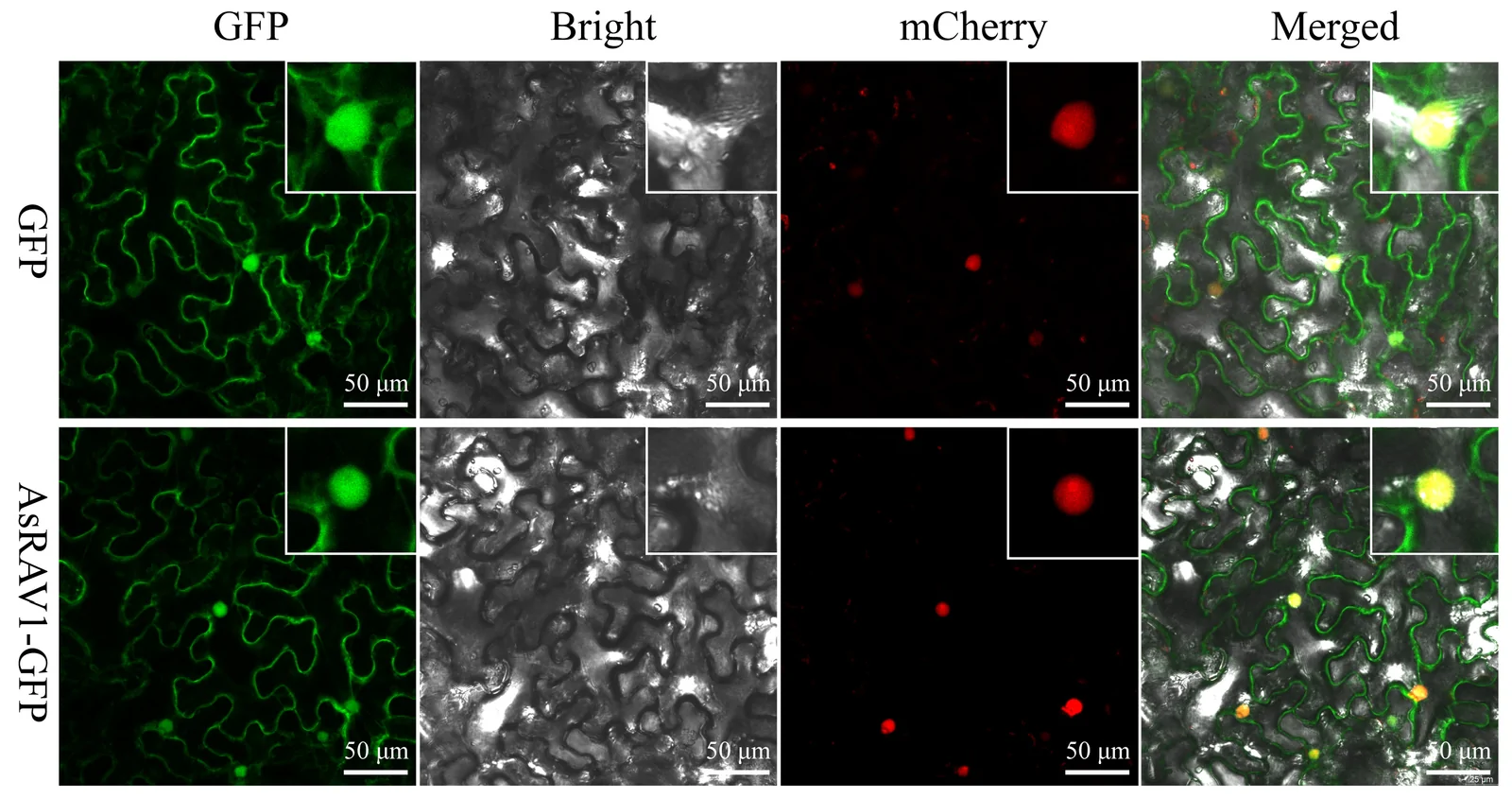
Subcellular localization of AsRAV1 in *N. benthamiana* leaves. AsRAV1-GFP (green) and the nuclear marker NLS-mCherry (red) were co-infiltrated into *N. benthamiana* leaves. GFP alone was used as a control. Scale bars = 50 μm.

Given that *AsRAV1* exhibited the highest expression level under PEG-simulated drought stress, it was selected for subcellular localization analysis. The *AsRAV1-GFP* fusion construct was transiently expressed in *N. benthamiana* leaf cells. Confocal microscopy revealed that the AsRAV1-GFP fusion protein was localized to both the nucleus and cytoplasm, confirming the dual subcellular localization of AsRAV1 (Figure 8).

## 4. Discussion

Genome-wide analyses of *RAV* genes have been performed in various plant species, including *A. thaliana* [34], *T. aestivum* [21], *O. sativa* [35], *Gossypium hirsutum* [36], *Glycine max* [11], *Cucumis sativus* [12], *Pyrus bretschneideri* [37], and *Capsicum annuum* [38]. In *A. thaliana*, a total of 13 *RAV* genes have been identified, of which only six (*TEM1*, *TEM2*, *RAV1*, *RAV1-like*, *RAV3*, and *RAV3-like*) possess both the B3 and AP2 domains [3,34]. Similarly, 15 *RAV* genes have been identified in rice, but only four members (*OsRAV8*, *OsRAV9*, *OsRAV11*, and *OsRAV12*) contain both domains [20,35]. However, little is known about the *RAV* gene family in oat. In this study, 12 *AsRAV* members were identified in oat through genome-wide analysis, all of which contained both AP2 and B3 domains. Phylogenetic analysis classified these 12 *AsRAV* genes into four distinct groups. Physicochemical property analysis revealed that members of Groups I, II, and IV were basic and likely unstable (pI > 7.00, instability index > 40), whereas Group III members (*AsRAV3*, *AsRAV7*, and *AsRAV10*) were acidic and likely stable (pI < 7.00, instability index < 40). Notably, Group III was the only group lacking motif 8, implying a potential structural and functional divergence of this group from the others.

Cultivated hexaploid oat has undergone whole-genome duplication and large-scale segmental duplication events during evolution [39]. Tandem duplication events are defined as chromosomal regions within 200 kb carrying two or more genes [40,41]. To investigate the evolutionary expansion of the *RAV* gene family in oat, we performed collinearity and synteny analyses. The results revealed that both tandem and segmental duplication events have contributed to the expansion of the *AsRAV* family. Synteny analysis identified 12 *AsRAV-OsRAV* orthologous pairs, 28 *AsRAV-TaRAV* orthologous pairs, 18 *AsRAV-HvRAV* orthologous pairs, 15 *AsRAV-SiRAV* orthologous pairs, and 27 *AsRAV-ZmRAV* orthologous pairs. In contrast, no syntenic pairs were found with *A. thaliana RAV* genes, suggesting limited detectable *RAV* synteny between oat and *A. thaliana*. This may be due to genome rearrangement, gene loss, or sequence divergence after the monocot-dicot split. Phylogenetic analysis revealed that *A. thaliana* RAV proteins formed a separate clade (Group V) distinct from monocot RAVs. Although monocot and dicot *RAV* genes are derived from a common ancestral lineage, the limited synteny conservation suggests that *RAV* genes may have undergone considerable divergence following the monocot–dicot split. Moreover, the *Ka/Ks* ratios of all orthologous and paralogous gene pairs were less than 1, indicating that these genes have undergone strong purifying selection and suggesting functional conservation during evolution.

RAV transcription factors have been reported to regulate drought stress responses in various plant species. For example, *NtRAV4* negatively regulates drought tolerance in tobacco by modulating stomatal behavior and activating antioxidants [42]. Similarly, *PgRAV-04* and *PgRAV_01* have been identified as negative regulators of drought tolerance in tobacco [43,44]. In contrast, *MeRAV5* positively regulates drought tolerance in cassava by modulating H_2_O_2_ and lignin accumulation through interaction with *MePOD* and *MeCAD15* [45]. However, the roles of *RAV* genes in oat drought response remain unknown. In this study, we investigated the expression patterns of *AsRAV* genes under drought stress using both transcriptomic and qRT-PCR analyses. Nine *AsRAV* genes were significantly up-regulated under PEG-simulated drought stress, with *AsRAV1* showing the highest induction level. These genes contain drought-related *cis*-elements (ABA, MYB-binding site, and MeJA). However, the frequency of these *cis*-elements was not correlated with the direction of expression change. These results suggest that *cis*-element prediction alone is not sufficient to infer expression patterns. Furthermore, sequence alignment against the rice genome showed that *AsRAV1* is most closely related to *OsRAV11* and *OsRAV12*, both of which have been functionally validated to play important roles in drought stress response in rice via knockout and overexpression assays [46]. We therefore speculate that *AsRAV1* may play a conserved role in the drought stress response in oat. Similar to MeRAV5 in cassava [45], AsRAV1 exhibited both nuclear and cytoplasmic localization. This dual distribution likely supports its functional versatility. As proposed for MeRAV5, the nuclear pool of AsRAV1 likely enables its role as a transcription factor to modulate downstream gene expression, whereas its cytoplasmic presence may facilitate interactions with cytosolic proteins and participate in additional signaling processes. Notably, this subcellular distribution pattern alone is insufficient to confirm its specific role in stress responses. Functional validation via gene silencing or overexpression of *AsRAV1* in oat is therefore required.

## 5. Conclusions

In conclusion, we systematically characterized 12 *AsRAV* genes in oat, which were classified into four groups. Tandem and segmental duplications have driven the expansion of the *AsRAV* gene family, and these genes have undergone strong purifying selection. The qRT-PCR analyses revealed that nine *AsRAV* genes were significantly induced by PEG-simulated drought stress, with *AsRAV1* showing the highest induction level and localized to both the nucleus and the cytoplasm. Collectively, these findings lay a foundation for understanding the *RAV* gene family in oat.

## Figures and Tables

**Table 1:** Characterization of AsRAV genes in oat.

Gene ID	Gene Name	Protein Length (aa)	MW (kDa)	pI	Instability Index	GRAVY	AP2 Domain	B3 Domain
AVESA.00010b.r2.1AG0006320.1	*AsRAV1*	376	40.14	9.45	46.13	−0.373	64–120	192–293
AVESA.00010b.r2.1AG0037940.1	*AsRAV2*	381	40.91	9.53	41.81	−0.346	64–120	192–295
AVESA.00010b.r2.1CG0111570.1	*AsRAV3*	327	35.37	5.89	31.55	−0.618	55–111	179–270
AVESA.00010b.r2.1CG0111580.1	*AsRAV4*	351	38.44	9.66	44.5	−0.436	60–116	174–264
AVESA.00010b.r2.1DG0157070.1	*AsRAV5*	384	41.12	9.46	43.87	−0.407	64–124	192–295
AVESA.00010b.r2.3AG0406080.1	*AsRAV6*	353	38.46	9.64	48.13	−0.461	61–116	174–264
AVESA.00010b.r2.3AG0406090.1	*AsRAV7*	321	34.88	5.89	32.27	−0.617	55–111	179–269
AVESA.00010b.r2.3AG0444190.1	*AsRAV8*	442	46.91	9.15	47.64	−0.398	132–188	263–354
AVESA.00010b.r2.3CG0453500.1	*AsRAV9*	350	38.02	9.31	42.59	−0.394	59–115	173–263
AVESA.00010b.r2.3CG0453510.1	*AsRAV10*	325	35.09	5.69	31.67	−0.531	55–115	178–268
AVESA.00010b.r2.3CG0497610.1	*AsRAV11*	397	41.60	9.17	41.5	−0.31	83–139	218–314
AVESA.00010b.r2.3DG0544780.1	*AsRAV12*	452	48.03	8.96	44.68	−0.366	145–201	273–364

## Data Availability

The original contributions presented in the study are included in the article and Supplementary Material; further inquiries can be directed to the corresponding author.

## Supplementary Materials

The following supporting information can be downloaded at: https://www.mdpi.com/article/10.3390/biology15191749/s1, Figure S1: Multiple sequence alignment of AsRAV proteins; Figure S2: Conserved motif logos of AsRAV proteins; Figure S3: Distribution of *cis*-acting elements in *AsRAV* gene promoters; Figure S4: Expression profiles of *AsRAV* genes based on RNA-seq data. Log2 FC were calculated at each time point (6, 12, 24, 48, and 72 h) relative to 0 h. The numbers above the bars indicate adjusted *P* values (padj); Table S1: Sequence information of RAV proteins in *A. thaliana*, *O. sativa*, *T. aestivum*, *H. vulgare, S. italica,* and *Z. mays*; Table S2: Primer sequences used in this study for *AsRAV* genes; Table S3: Sequence information of AsRAV proteins in oat; Table S4: Segmental and tandem duplicated *AsRAV* gene pairs; Table S5: *Ka* and *Ks* values and *Ka/Ks* ratios of *AsRAV* duplicated gene pairs; Table S6: *Ka* and *Ks* values and *Ka/Ks* ratios of orthologous gene pairs between *A. sativa* and *O. sativa*; Table S7: *Ka* and *Ks* values and *Ka/Ks* ratios of orthologous gene pairs between *A. sativa* and *T. aestivum*; Table S8: *Ka* and *Ks* values and *Ka/Ks* ratios of orthologous gene pairs between *A. sativa* and *H. vulgare*; Table S9: *Ka* and *Ks* values and *Ka/Ks* ratios of orthologous gene pairs between *A. sativa* and *S. italica*; Table S10: *Ka* and *Ks* values and *Ka/Ks* ratios of orthologous gene pairs between *A. sativa* and *Z. mays*; Table S11: Flanking genes of *AsRAV* genes within syntenic blocks across five grass species; Table S12: Transcriptomic expression profiles of the *AsRAV* genes under drought stress.

## References

[1] Zhao J., Huang M., Liu J., Cai J.Y., He Y.H., Zhao W.J., Liu C.Z., Wu Y.J. (2025). Pepper (*Capsicum annuum* L.) AP2/ERF transcription factor, *CaERF2* enhances salt stress tolerance through ROS scavenging. Theor. Appl. Genet..

[2] Gao J., Ma G.J., Chen J.J., Gichovi B., Cao L.W., Liu Z.H., Chen L. (2024). The *B3* gene family in *Medicago truncatula*: Genome-wide identification and the response to salt stress. Plant Physiol. Biochem..

[3] Matías-Hernández L., Aguilar-Jaramillo A.E., Marín-González E., Suárez-López P., Pelaz S. (2014). *RAV* genes: Regulation of floral induction and beyond. Ann. Bot..

[4] Cheng H., Wang Q.G., Zhang Z.X., Cheng P.L., Song A.P., Zhou L.J., Wang L.K., Chen S.M., Chen F.D., Jiang J.F. (2023). The RAV transcription factor TEMPRANILLO1 involved in ethylene-mediated delay of chrysanthemum flowering. Plant J..

[5] Kagaya Y., Ohmiya K., Hattori T. (1999). RAV1, a novel DNA-binding protein, binds to bipartite recognition sequence through two distinct DNA-binding domains uniquely found in higher plants. Nucleic Acids Res..

[6] Wang P., Yan Y., Lu Y., Liu G.Y., Liu J.P., Shi H.T. (2022). The co-modulation of RAV transcription factors in ROS burst and extensive transcriptional reprogramming underlies disease resistance in cassava. Plant Cell Rep..

[7] Zhang Z.Y., Shi Y.N., Ma Y.C., Yang X.F., Yin X.R., Zhang Y.Y., Xiao Y.W., Liu W.L., Li Y.D., Li S.J. (2020). The strawberry transcription factor FaRAV1 positively regulates anthocyanin accumulation by activation of FaMYB10 and anthocyanin pathway genes. Plant Biotechnol. J..

[8] Wang S.M., Guo T., Zhang S.L., Yang H., Li L., Yang Q.C., Quan J.P., Long R.C. (2025). Functional identification of *Medicago truncatula MtRAV1* in regulating growth and development. J. Integr. Agric..

[9] Xue Y.G., Zhang Y.T., Shan J.M., Ji Y.J., Zhang X.M., Li W.B., Li D.M., Zhao L. (2022). Growth repressor GmRAV binds to the *GmGA3ox* promoter to negatively regulate plant height development in soybean. Int. J. Mol. Sci..

[10] Hu H.M., Tian S., Xie G.H., Liu R., Wang N.N., Li S.S., He Y.H., Du J.M. (2021). TEM1 combinatorially binds to *FLOWERING LOCUS T* and recruits a Polycomb factor to repress the floral transition in *Arabidopsis*. Proc. Natl. Acad. Sci. USA.

[11] Zhao S.P., Xu Z.S., Zheng W.J., Zhao W., Wang Y.X., Yu T.F., Chen M., Zhou Y.B., Min D.H., Ma Y.Z. (2017). Genome-wide analysis of the *RAV* family in soybean and functional identification of *GmRAV-03* involvement in salt and drought stresses and exogenous ABA treatment. Front. Plant Sci..

[12] Li J.L., Song C.Y., Li H.M., Wang S.Q., Hu L.Y., Yin Y.L., Wang Z.H., He W.X. (2023). Comprehensive analysis of cucumber *RAV* family genes and functional characterization of *CsRAV1* in salt and ABA tolerance in cucumber. Front. Plant Sci..

[13] Cui T.Z., Zang S.J., Sun X.L., Zhang J., Su Y.C., Wang D.J., Wu G.R., Chen R.Q., Que Y.X., Lin Q. (2024). Molecular identification and functional characterization of a transcription factor GeRAV1 from *Gelsemium elegans*. BMC Genom..

[14] Chen X.L., Shang L.N., Zhang M.H., Wen S.Q., Li Y., Li M.K., Jian H.J., Lv D.Q. (2026). Transcription factor StHRAV1 negatively regulates thermotolerance by modulating ROS accumulation in potato. J. Agric. Food Chem..

[15] Alemayehu G.F., Forsido S.F., Tola Y.B., Amare E. (2023). Nutritional and phytochemical composition and associated health benefits of oat (*Avena sativa*) grains and oat-based fermented food products. Sci. World J..

[16] Kim I.S., Hwang C.W., Yang W.S., Kim C.H. (2021). Multiple antioxidative and bioactive molecules of oats (*Avena sativa* L.) in human health. Antioxidants.

[17] Zhao B.P., Ma B.L., Hu Y.G., Liu J.H. (2021). Source–sink adjustment: A mechanistic understanding of the timing and severity of drought stress on photosynthesis and grain yields of two contrasting oat (*Avena sativa* L.) genotypes. J. Plant Growth Regul..

[18] Zhang X.J., Liu W.T., Lv Y.C., Li T.L., Tang J.Z., Yang X.H., Bai J., Jin X., Zhou H.T. (2022). Effects of drought stress during critical periods on the photosynthetic characteristics and production performance of Naked oat (*Avena nuda* L.). Sci. Rep..

[19] Kamal N., Tsardakas Renhuldt N., Bentzer J., Gundlach H., Haberer G., Juhász A., Lux T., Bose U., Tye-Din J.A., Lang D. (2022). The mosaic oat genome gives insights into a uniquely healthy cereal crop. Nature.

[20] Osnato M., Matias-Hernandez L., Aguilar-Jaramillo A.E., Kater M.M., Pelaz S. (2020). Genes of the *RAV* family control heading date and carpel development in rice. Plant Physiol..

[21] Luo Y.X., Chen S.K., Wang P.D., Peng D., Zhang X., Li H.F., Feng C.Z. (2022). Genome-wide analysis of the *RAV* gene family in wheat and functional identification of *TaRAV1* in salt stress. Int. J. Mol. Sci..

[22] Paysan-Lafosse T., Andreeva A., Blum M., Chuguransky S.R., Grego T., Pinto B.L., Salazar G.A., Bileschi M.L., Llinares-López F., Meng-Papaxanthos L. (2025). The Pfam protein families database: Embracing AI/ML. Nucleic Acids Res..

[23] Wang J.Y., Chitsaz F., Derbyshire M.K., Gonzales N.R., Gwadz M., Lu S.N., Marchler G.H., Song J.S., Thanki N., Yamashita R.A. (2023). The conserved domain database in 2023. Nucleic Acids Res..

[24] Letunic I., Bork P. (2026). SMART v10: Three decades of the protein domain annotation resource. Nucleic Acids Res..

[25] Chen C.J., Wu Y., Li J.W., Wang X., Zeng Z.H., Xu J., Liu Y.L., Feng J.T., Chen H., He Y.H. (2023). TBtools-II: A “one for all, all for one” bioinformatics platform for biological big-data mining. Mol. Plant.

[26] Bailey T.L., Johnson J., Grant C.E., Noble W.S. (2015). The MEME suite. Nucleic Acids Res..

[27] Lescot M., Déhais P., Thijs G., Marchal K., Moreau Y., van de Peer Y., Rouzé P., Rombauts S. (2002). PlantCARE, a database of plant *cis*-acting regulatory elements and a portal to tools for in silico analysis of promoter sequences. Nucleic Acids Res..

[28] Letunic I., Bork P. (2024). Interactive Tree of Life (iTOL) v6: Recent updates to the phylogenetic tree display and annotation tool. Nucleic Acids Res..

[29] Xu S.R., Ji X.J., Sai F.M., Ma M.C., Liu Z., Zhang L.J., Liu L.L. (2026). Comprehensive investigation of *GRF* transcription factors and associated responses to drought stress in oat (*Avena sativa*). Plants.

[30] Chen Y., Li A.X., Yun P., Chen Q., Pan D.Y., Guo R., Zhang H., Ahmed H.A.I., Hu H.Y., Peng Y.Y. (2024). Genome-wide analysis of *MYB* transcription factor family and *AsMYB1R* subfamily contribution to ROS homeostasis regulation in *Avena sativa* under PEG-induced drought stress. BMC Plant Biol..

[31] Michel B.E., Kaufmann M.R. (1973). The osmotic potential of polyethylene glycol 6000. Plant Physiol..

[32] Zhao D.B., Wei L.X., Guo J.J., Guo Z.H., Guo L.H., Gao J.S., Cui H.N., Tai R.J., Guan P.Y., Zhang L. (2026). Identification and expression profiling of *LSD* genes reveal their role in developmental and abiotic stress conditions in maize. Front. Plant Sci..

[33] Gao X.Y., Zhang J.Q., Zhang X.J., Zhou J., Jiang Z.S., Huang P., Tang Z.B., Bao Y.M., Cheng J.P., Tang H.J. (2019). Rice qGL3/OsPPKL1 functions with the GSK3/SHAGGY-like kinase OsGSK3 to modulate brassinosteroid signaling. Plant Cell.

[34] Romanel E.A., Schrago C.G., Couñago R.M., Russo C.A., Alves-Ferreira M. (2009). Evolution of the B3 DNA binding superfamily: New insights into *REM* family gene diversification. PLoS ONE.

[35] Chen C.H., Li Y.J., Zhang H.H., Ma Q., Wei Z.Y., Chen J.P., Sun Z.T. (2021). Genome-wide analysis of the *RAV* transcription factor genes in rice reveals their response patterns to hormones and virus infection. Viruses.

[36] Kabir N., Lin H., Kong X.H., Liu L., Qanmber G., Wang Y.X., Zhang L., Sun Z.J., Yang Z.R., Yu Y. (2021). Identification, evolutionary analysis and functional diversification of *RAV* gene family in cotton (*G. hirsutum* L.). Planta.

[37] Liu J.L., Deng Z.W., Liang C.L., Sun H.W., Li D.L., Song J.K., Zhang S.L., Wang R. (2021). Genome-wide analysis of *RAV* transcription factors and functional characterization of anthocyanin-biosynthesis-related *RAV* genes in pear. Int. J. Mol. Sci..

[38] Pei M.K., Yang P., Li J., Wang Y.Z., Li J., Xu H.J., Li J. (2024). Comprehensive analysis of pepper (*Capsicum annuum*) *RAV* genes family and functional identification of *CaRAV1* under chilling stress. BMC Genom..

[39] He Q., Li W., Miao Y.Q., Wang Y., Liu N.K., Liu J.N., Li T., Xiao Y., Zhang H.Y., Wang Y.R. (2024). The near-complete genome assembly of hexaploid wild oat reveals its genome evolution and divergence with cultivated oats. Nat. Plants.

[40] Holub E.B. (2001). The arms race is ancient history in *Arabidopsis*, the wildflower. Nat. Rev. Genet..

[41] Xie T., Chen C.J., Li C.H., Liu J.R., Liu C.Y., He Y.H. (2018). Genome-wide investigation of *WRKY* gene family in pineapple: Evolution and expression profiles during development and stress. BMC Genom..

[42] Gao Y., Yang J., Duan W.J., Ma X.H., Qu L.L., Xu Z.C., Yang Y.X., Xu J.Y. (2022). *NtRAV4* negatively regulates drought tolerance in *Nicotiana tabacum* by enhancing antioxidant capacity and defence system. Plant Cell Rep..

[43] Wang Y.H., Ye X., Zhao B.Y., Wang W.J., Zhou Z.F., Zhang X.Q., Du J., Song J.L., Huang X.L., Ouyang K.X. (2024). Comprehensive analysis of *B3* family genes in pearl millet (*Pennisetum glaucum*) and the negative regulator role of *PgRAV-04* in drought tolerance. Front. Plant Sci..

[44] Wang Y.H., Zhao B.Y., Ye X., Du J., Song J.L., Wang W.J., Huang X.L., Ouyang K.X., Zhang X.Q., Liao F.X. (2024). Genome-wide analysis of the *AP2/ERF* gene family in *Pennisetum glaucum* and the negative role of *PgRAV01* in drought tolerance. Plant Physiol. Biochem..

[45] Yan Y., Wang P., Lu Y., Bai Y.J., Wei Y.X., Liu G.Y., Shi H.T. (2021). *MeRAV5* promotes drought stress resistance in cassava by modulating hydrogen peroxide and lignin accumulation. Plant J..

[46] Zhang Y., Ikram M., Khan N., Zhao N., Derakhshani B., Usman B., Wang H.F. (2025). Machine learning and functional validation identify *OsRAV11/12* as negative regulators of drought tolerance and early flowering in rice. Int. J. Biol. Macromol..

