# Supplementary material for: Comprehensive Analysis of the RAV Gene Family and Its Expression Patterns Under PEG-Simulated Drought Stress in Avena sativa
Source: Biology (Basel). 2026 Oct 2;15(19):1749. doi: 10.3390/biology15191749 (PMC13663434; doi:10.3390/biology15191749)
Supplement: Supplementary file 1 [file biology-15-01749-s001.zip › Supplementary figures S1-S4.pdf]

Supplementary Material

Supplementary Figures

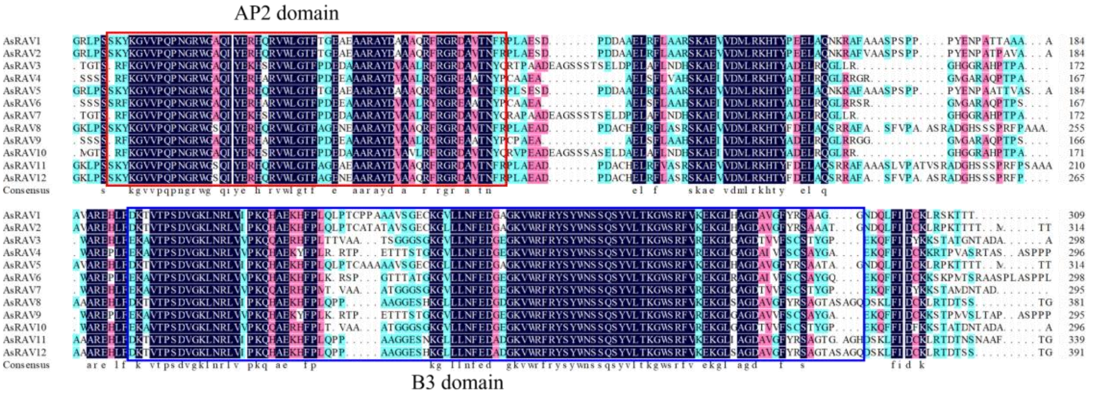

Figure S1. Multiple sequence alignment of AsRAV proteins.

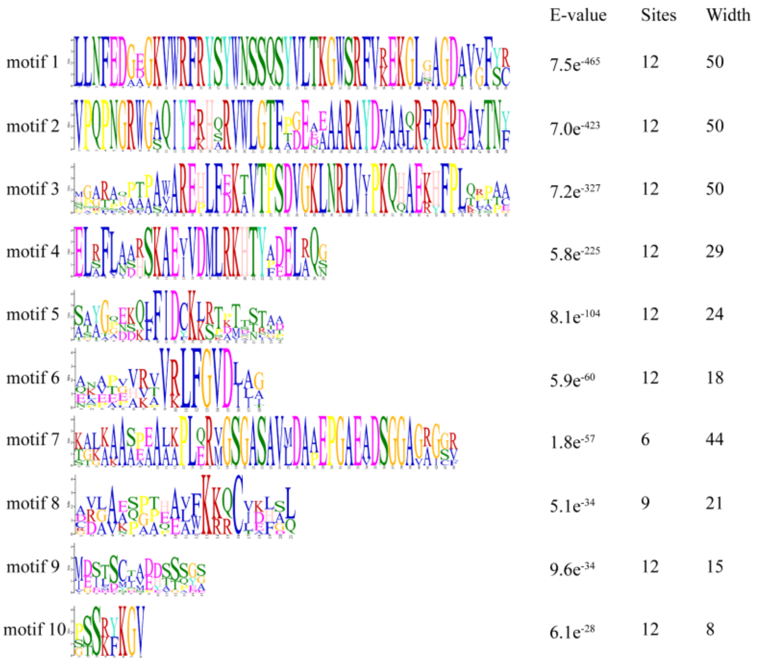

Figure S2. Conserved motif logos of AsRAV proteins.

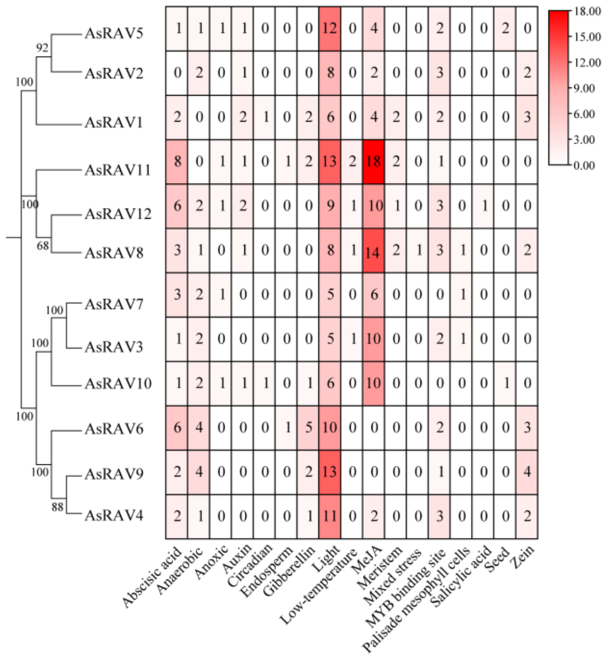

Figure S3. Distribution of cis-acting elements in *AsRAV* gene promoters.

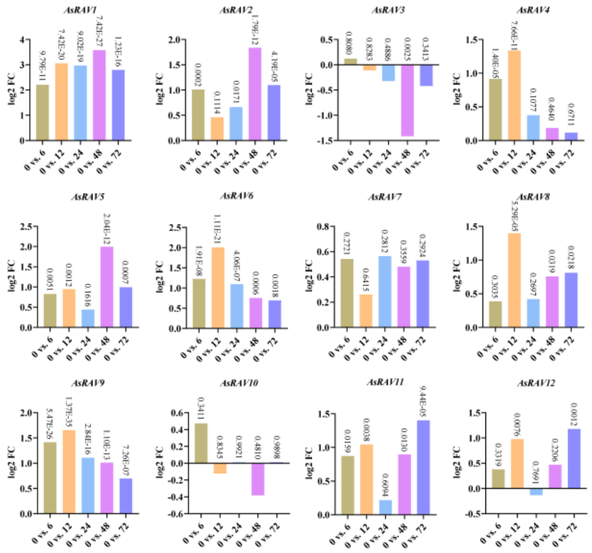

Figure S4. Expression profiles of *AsRAV* genes based on RNA-seq data. Log2 FC were calculated at each time point (6, 12, 24, 48, and 72 h) relative to 0 h. The numbers above the bars indicate adjusted *P* values (padj).
